# Congenital Anophthalmia and Binocular Neonatal Enucleation Differently Affect the Proteome of Primary and Secondary Visual Cortices in Mice

**DOI:** 10.1371/journal.pone.0159320

**Published:** 2016-07-13

**Authors:** Marie-Eve Laramée, Katrien Smolders, Tjing-Tjing Hu, Gilles Bronchti, Denis Boire, Lutgarde Arckens

**Affiliations:** 1 Laboratory of Neuroplasticity and Neuroproteomics, Katholieke Universiteit Leuven, 3000, Leuven, Belgium; 2 Département d’anatomie, Université du Québec à Trois-Rivières, Québec, Canada; 3 École d’optométrie, Université de Montréal, Québec, Canada; Doheny Eye Institute and Keck School of Medicine of the University of Southern California, UNITED STATES

## Abstract

In blind individuals, visually deprived occipital areas are activated by non-visual stimuli. The extent of this cross-modal activation depends on the age at onset of blindness. Cross-modal inputs have access to several anatomical pathways to reactivate deprived visual areas. Ectopic cross-modal subcortical connections have been shown in anophthalmic animals but not in animals deprived of sight at a later age. Direct and indirect cross-modal cortical connections toward visual areas could also be involved, yet the number of neurons implicated is similar between blind mice and sighted controls. Changes at the axon terminal, dendritic spine or synaptic level are therefore expected upon loss of visual inputs. Here, the proteome of V1, V2M and V2L from P0-enucleated, anophthalmic and sighted mice, sharing a common genetic background (C57BL/6J x ZRDCT/An), was investigated by 2-D DIGE and Western analyses to identify molecular adaptations to enucleation and/or anophthalmia. Few proteins were differentially expressed in enucleated or anophthalmic mice in comparison to sighted mice. The loss of sight affected three pathways: metabolism, synaptic transmission and morphogenesis. Most changes were detected in V1, followed by V2M. Overall, cross-modal adaptations could be promoted in both models of early blindness but not through the exact same molecular strategy. A lower metabolic activity observed in visual areas of blind mice suggests that even if cross-modal inputs reactivate visual areas, they could remain suboptimally processed.

## Introduction

The loss of visual inputs results in the activation of occipital areas by non-visual stimuli. This has been observed in blind humans (see [1] for Review), as well as in visually deprived mice [2–4], rats [5–7], hamsters [8], opossum [9, 10], blind mole rat [11, 12] and cats [13, 14]. Both striate and extrastriate areas are activated in the early blind during a non-visual task, whereas only extrastriate areas are activated in the late blind [15–19]. Similar results were found in macaques [20], rats [7, 21, 22] and mice [2, 3].

Ectopic subcortical connections and the potentiation of direct and indirect cortico-cortical inputs could be involved in the cross-modal activation of occipital areas in the blind. Cross-modal subcortical connections do not naturally occur in sighted animals but they have been described in anophthalmic mice [2, 3, 23] and in the blind mole rat [24]. In contrast, direct cross-modal cortico-cortical connections have been observed in sighted humans [25], non-human primates [26–28], mice [4, 29, 30], gerbils [31], prairie voles [32] and opossums [33] and could be potentiated after the loss of visual inputs. Indirect cortico-cortical connections through secondary visual areas [34–38] or higher order multisensory areas (see [39] for Review) could also take part in this process. Very few differences in cortico-cortical cross-modal inputs were found between blind and sighted mice [29] but fine structural reorganization could occur. Such morphological changes would require the participation of several key proteins involved in axon and dendrite outgrowth, pruning, axonal transport and establishment of new synapses. Furthermore, the pre-existent cross-modal connections, which are subthreshold in sighted subjects [40–42], could be unmasked in the blind by changes in the balance between excitation and inhibition [43]. This could be achieved by Hebbian and homeostatic plasticity mechanisms which depend on different processes namely trafficking of postsynaptic receptors, anchoring of these receptors at the postsynaptic density, changes in their subunit composition and phosphorylation status of their subunits (see [44] for Review).

In the present study, we screened for the long-term molecular footprint of early loss of sight in visual cortex of binocularly enucleated and congenital anophthalmic mice. The changes in protein expression were assessed in V1, V2M and V2L using two-dimensional differential gel electrophoresis (2-D DIGE) and Western analyses.

## Material and Methods

### Animals

All animal procedures were approved by the University Animal Care Committee of the Université du Québec à Trois-Rivières and were carried out in accordance with the guidelines set out by the Canadian Council on Animal Care. Animal health was monitored on a daily basis by the animal caretakers from the animal facility of the Université du Québec à Trois-Rivières. None of the animals included in this study showed signs of pain or discomfort.

In order for all mice to have the same genetic background, mice were cross-bred from C57BL/6J and ZRDCT/An mice. By backcrossing the C57BL/6J-ZRDCT/An pups with ZRDCT/An mice up to generation F4, we obtained hybrid mice with half of the pups born with normal eyes (heterozygous) and half of them anophthalmic (homozygous). Half the pups born with eyes were enucleated within the first 24 hours. All mice used in this study were P60 (+/- 1 day). They were sighted controls (n = 5), bilaterally enucleated at P0 (n = 5) and congenitally anophthalmic (n = 5) littermates.

### Brain Extraction and Tissue Punching

At P60, mice were anesthetized with isoflurane. The brains were extracted and flash-frozen in -40°C 2-methylbutane (Sigma-Aldrich) and stored at -80°C. Serial coronal sections of 3x 100μm thick, for protein extraction, and of 1x 25μm thick, for Nissl staining, were cut on a Microm HM 500 OM cryostat and kept at -80°C and -20°C, respectively.

All 25μm sections were processed for Nissl staining according to standard procedures. Pictures of stained sections were acquired with a Zeiss AxioImager Z1 motorized microscope (5x objective, 0.16 NA) coupled to an AxioCam MRm CCD camera and AxioVision software (Carl Zeiss Benelux). Areal cytoarchitectonic features were afterward used to draw the borders of all the areas of interest [45, 46].

Using the delineated pictures of Nissl stained sections as an atlas for each brain, tissue from V1, V2M and V2L was dissected and collected from both hemispheres on an average of fifteen 100μm thick sections. Brain tissue was transferred to 100μl ice-cold lysis buffer, containing 7M urea, 2M thiourea, 4% w/v 3-[(3-cholamidopropyl)dimethylammonio]-1-propanesulfonate (Sigma-Aldrich), 1% w/v dithiothreitol (DTT) (Serva), 40mM Tris Base (ICN; Aurora), and Complete Protease Inhibitor Cocktail (Roche Diagnostics). Homogenization of tissue was then performed on ice, followed by a brief 13000g centrifugation, sonication and complete solubilization of the proteins for 1h at room temperature. Proteins were then briefly sonicated again before being centrifuged for 20min at 13000g at 4°C to allow for cell debris precipitation. The supernatant was dialyzed against milli-Q water for 2h to remove residual salt using a membrane with a 500-Da cutoff (Spectra/Por, Biotech, Omnilabo). Protein concentrations were afterward determined with the Qubit^TM^ Quantification Platform (Invitrogen) using a Qubit^TM^ fluorometer (Invitrogen).

### Two-Dimensional Difference Gel Electrophoresis

The two-dimensional difference gel electrophoresis (2-D DIGE) was used as it allows for the separation of the protein samples in two dimensions based (1) on their isoelectric point and (2) on their molecular weight [47]. This allows for the detection of post-translational changes and for the separation of different protein isoforms in distinct spots on the gel.

The fluorescent cyanine dyes, Cy2 (Cy2), propyl-Cy3 (Cy3) and methyl-Cy5 (Cy5) were in-house synthesized [47–49]. All other chemicals were purchased from GE Healthcare, unless otherwise mentioned.

Pre-cast Immobiline DryStrips (24cm, pH 3–11 nonlinear) were rehydrated overnight in DeStreak Rehydratation Solution containing 0.5% v/v immobilized pH gradient (IPG) buffer in a reswelling tray covered with paraffin oil (Merck). The next day, 50μg of protein of each sample was randomly labeled with either Cy3 or Cy5. Equal fractions of all samples were pooled and 50μg of this pool was labeled with Cy2 to serve as an internal standard. The minimal amount of dye that gave a maximum number of spots and the highest signal-to-noise ratio was set to approximately 200pmol, as described previously [48, 49]. During the labeling process, the samples were incubated for 30min on ice and in the dark. The reaction was subsequently stopped by adding 1μl of lysine (10mM; Merck). The samples were again incubated on ice for 15min in the dark. The Cy2-, Cy3- and Cy5-labeled fractions were then mixed together, and an equal volume of lysis buffer solution was added. Isoelectric focusing (IEF) was performed on an Ettan IPGphor Cup Loading Manifold system, according to manufacturer’s instructions. Run conditions were 300V for 3h, 600V for 3h, followed by a 6h gradient to 1000V, a 3h gradient to 8000V and 8h at 8000V for a total of 40-50kVh (at 0.05mA/strip). After IEF, the strips were equilibrated twice in equilibration buffer (6M urea, 34.5% v/v glycerol and 10% w/v SDS in Tris-HCl buffer [1.5M, pH 8.8]) initially containing DTT (1% w/v) and then 4.5% w/v iodoacetamide (Sigma-Aldrich). Both incubations steps were 15min. Electrophoresis of the IPG strips was then done on 1.5mm thick SDS-polyacrylamide gels (12.5% T; 2.6% C) in the Ettan DALT twelve system for 30min at 30mA and 24h at 15mA/gel, at 13°C.

### Image Analysis and Statistics

Gels were scanned with the Ettan DIGE Imager (software 1.0; GE Healthcare) and gel image triplets (Cy2, Cy3 and Cy5) comprising the CyDye-labeled proteins were generated. Quantitative analysis was carried out with the DeCyder 2D difference analysis software (Version 7.0; GE Healthcare). Spot detection and matching was performed automatically with the DeCyder Batch processor. The gel-to-gel matching was verified manually followed by statistical analysis of protein abundance change between samples in the biological variation analysis (BVA) module embedded in the DeCyder Software [50–52]. Spots of interest with *P <* 0.05 were selected for identification. Spots with a p-value of *P <* 0.1 and with a change in expression ratio of at least +/- 30% (Average ratio, AR > |1.3|) in fluorescence level were also selected to increase the data set for the pathway analysis.

### Protein Identification

Preparative gels were ran under the same conditions as described above, except for the last step of the IEF that ran for 15h at 8000V, for a total of 70-80kVh. Each gel was loaded with 1. 7mg of protein from the pool sample, from which only a 50μg fraction was labeled with Cy3. Glass plates were pretreated with BindSilane, and two reference markers were applied to enable automatic spot picking. The preparative gels were scanned in the Ettan DIGE Imager to obtain an image of the Cy3 signal. The total protein load was then visualized with Lava Purple (Fluorotechnics) fluorescent stain, according to the manufacturer’s instructions and scanned again. Matching with the analytical gels was carried out automatically with manual correction by the BVA module of the Decyder software (GE Healthcare). A pick list of the proteins of interest was generated and imported into the Spot Picker Version 1.20 software that controls the Ettan Spot Picker (GE Healthcare). Identical spots from two different gels were pooled.

In collaboration with the Luxembourg Institute of Science and Technology, MS and MS/MS spectra were acquired using a 5800 MALDI TOF-TOF (ABSciex, Sunnyvale, CA, U.S.A.), calibrated using the 4700 peptide mass calibration kit (Applied Biosystems). Proteins were identified by searching against the NCBI database, limited to the taxonomy of Rodents (downloaded 2012.06.04, 316675 sequences), using an in-house MASCOT server (version 2.3.0 Matrix Science, www.matrixscience.com, London, U.K.). Searches were carried out defining trypsin as cleavage agent and allowing for 2 missed cleavages. A mass window of 150 ppm was tolerated for the precursor mass and 0.75 Da for fragment ion masses. The search parameters allowed for carboxymethylation of cysteine as fixed modification and oxidation of methionine and of tryptophan (double oxidation, and kynurenin formation) as variable modifications. Proteins were considered identified when two, none overlapping, individual peptides surpassed the peptide score threshold. When this criterion was not met, additional precursors were selected and searched using the above-described parameters. N-terminal acetylation was found by changing the cleavage to semi-tryptic and adding N-terminal acetyl as variable modification.

Identified proteins were loaded into the QIAGEN’s Ingenuity Pathway Analysis (IPA®, QIAGEN Redwood City, www.qiagen.com/ingenuity) software to identify the most relevant canonical pathways affected by enucleation or anophthalmia. The significant IPA Canonical pathways were identified with a Benjamini-Hochberg correction and a p- value of 0.05 was considered statistically relevant.

### Western blotting

Western blot experiments were used to verify the expression of several proteins identified by the 2-D DIGE analysis. The same samples prepared for 2-D DIGE were also used for Western blotting. The expression levels of other interesting proteins, associated to a pathway identified by the 2-D DIGE analysis, were also assessed by Western blot analysis. The list of proteins, quantities of protein loaded on the gel and information about the primary antibody are listed in Table 1. For each protein, dilution series were performed (2.5 to 30 μg) using the pool sample to determine the optimal protein load that gave the best signal-to-noise ratio while being in the linear range of the standard curve (Table 1).

**Table 1:** List of antibodies for western blot analyses.

Protein	Qty (μg)	Supplier**(Cat. number)**	RRID	Clonality	Host	Dilution	Blot
Alpha-Internexin	15	NovusBiological (NB300-139)	AB_10001006	Polyclonal	Rabbit	1: 5 000	Nitro
Beta-II-Tubulin	10	Abcam (ab28036)	AB_727047	Monoclonal	Mouse	1: 1 000	PVDF
Collapsin response mediator protein-2	15	M.D. Y. Ihara (University of Tokyo)	Ihara et al. 1999 (gift)	Polyclonal	Mouse	1: 50 000	Nitro
Collapsin response mediator protein-4	20	Chemicon (Ab 5454)	AB_91876	Polyclonal	Rabbit	1: 5 000	Nitro
Glutamate decarboxylase 65 kDa	15	Abcam (Ab55412)	AB_880148	Polyclonal	Rabbit	1: 1 000	PVDF
Glutamate decarboxylase 67 kDa	15	Abcam (Ab55412)	AB_880148	Polyclonal	Rabbit	1: 1 000	PVDF
Glutamate receptor AMPA 2	10	Millipore (MAB397)	AB_2113875	Monoclonal	Mouse	1: 500	PVDF
Glutamine synthetase	10	Millipore (MAB302)	AB_2110656	Monoclonal	Mouse	1: 2 000	PVDF
Neurofilament low	10	Millipore (MAB1615)	AB_94285	Monoclonal	Mouse	1: 5 000	PVDF
Synapsin-2a	7.5	Abcam (Ab61406)	AB_2042983	Polyclonal	Rabbit	1: 4 000	Nitro
Synapsin-2b	7.5	Abcam (Ab61406)	AB_2042983	Polyclonal	Rabbit	1: 4 000	Nitro
Syntaxin binding protein 1	10	ThermoScientific (PA1-742)	AB_325854	Polyclonal	Rabbit	1: 2 000	PVDF
Vesicular Glutamate Transporter 1	10	Millipore (MAB5502)	AB_262185	Monoclonal	Mouse	1: 2 000	PVDF
Vesicular Glutamate Transporter 2	10	Millipore (MAB5504)	AB_2187552	Monoclonal	Mouse	1: 500	PVDF

Nitrocellulose (Nitro), Polyvinylidene fluoride (PVDF), Quantity (Qty) and Research Resource Identification (RRID).

Protein samples were loaded onto a 4–12% Bis-Tris NuPage gel (BioRad). Electrophoresis was carried out (200V, 200mA, 25W) and blotting was performed using the Trans-Blot Turbo Transfer System (BioRad). Polyvinylidene fluoride (PVDF) or nitrocellulose membranes (Table 1) were afterward rinsed for 1–2 hrs in a 5% blocking buffer solution (Amersham ECL blocking solution, GE Healthcare). Membranes were incubated in the antibody solution overnight at room temperature (Table 1). The next day, blots were quickly rinsed in Tris-Saline solution before being placed in the secondary antibody solution for 30min. Secondary antibodies were either horseradish peroxidase-conjugated goat anti-mouse (GaM-HRP, 1: 50 000, Dako) or goat anti-rabbit (GaR-HRP, 1: 50 000, Dako). Immunoreactivity was visualized using chemiluminescence detection (SuperSignal West Dura Extended duration substrate, Thermo Scientific) on ECL hyperfilm (GE Healthcare) and imaged using the ChemiDoc MP Imaging system (BioRad).

The protein bands were semi-quantitatively evaluated by densitometry using the ImageLab software (BioRad). Optical density values were normalized with respect to the pool sample and with respect to the total protein stain to correct for inter and intra gel variability [53]. The total protein stain was carried out using a LavaPurple staining (Fluorotechnics) or a Coomassie Blue staining. All experiments were duplicated and the average values of the two runs were calculated for each sample. A one-way ANOVA with a LSD post-hoc test was performed using the averaged value for each sample. The significance level was set to *P* < 0.05. Statistical analyses were performed using SPSS 22 for Windows (IBM).

## Results

### Protein expression is affected in enucleated and anophthalmic mice

**Fig 1:**
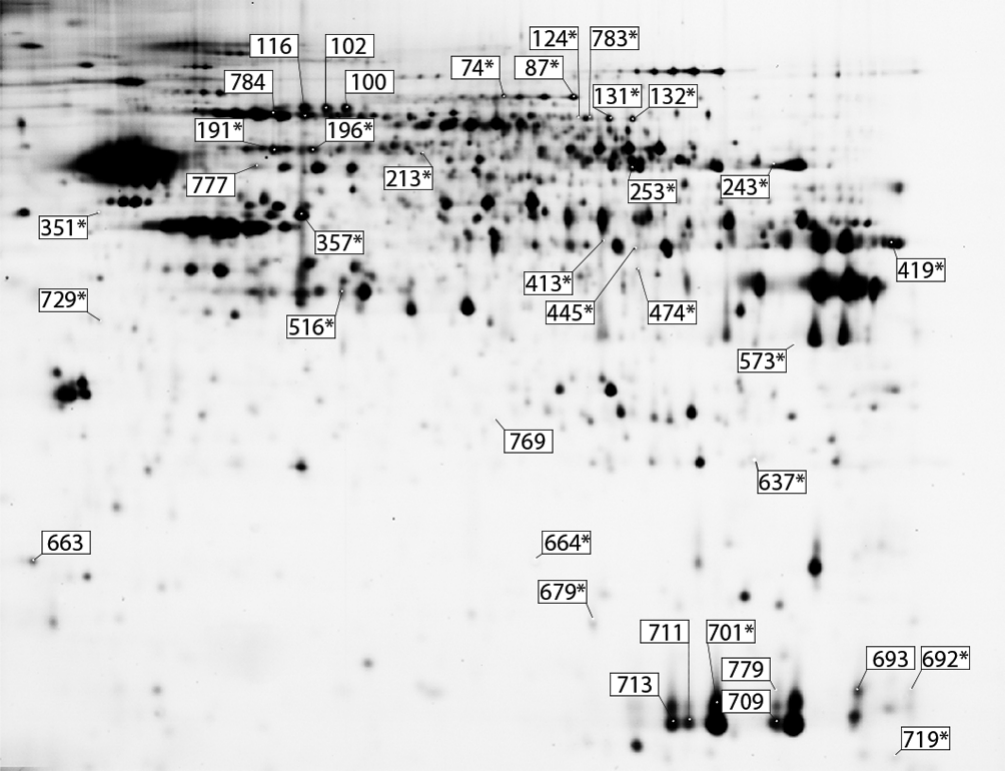
Spots with differential expression. 38 spots were differently expressed in enucleated or anophthalmic mice with respect to sighted mice. * spots with *P* < 0.05. Other spots with *P* < 0.1 and Average Ratio > |1.30|.

2-D DIGE was used as a screening technique to identify molecular pathways affected by neonatal binocular enucleation and/or congenital anophthalmia in V1, V2M and V2L of adult mice. A total of 2257 spots were identified and matched between the gels, with a mean of 752 spots per cortical area (V1: 792; V2M: 734 and V2L: 731). Among these, only 25 spots were found to be significantly different with respect to sighted control mice (17 in V1, 4 in V2M and 4 in V2L) when a significance level of *P <* 0.05 was chosen (Fig 1 and Table 2). This number was surprisingly low. To increase the data set in order to perform a pathway analysis, spots that displayed a change in expression ratio of at least +/- 30% in expression level (AR > |1.30|) with a significance level of *P <* 0.1 were also included in the analysis. With these parameters, a total of 38 spots displayed significant differences in expression levels in the visual areas of blind mice (Fig 1 and Table 2). The majority of these changes were observed in V1 (18 spots). Loss of sight affected the expression level of 14 spots in V2M, and 6 spots in V2L. Expression levels could be up- or down-regulated in V1, mostly up-regulated in V2L and always down-regulated in V2M (Table 3). For the spots showing significant changes in expression levels, consistent modulations were found between enucleated and anophthalmic mice.

**Table 2:** Identifications of differently expressed spots.

*P* value	SPOT	Mascot value	Accession number (GI number)	Identification
*P* <	74	129	40254595	collapsin response mediator protein 2
0.05	74	109	148702256	N-ethylmaleimide sensitive fusion protein, isoform CRA_a
	87	799	31543349	vesicle-fusing ATPase
	124			NO ID
	131	658	6981602	syntaxin-binding protein 1
	132	713	6981602	syntaxin-binding protein 1
	191	862	183396771	60 kDa heat shock protein, mitochondrial
	191	304	148539957	alpha-internexin
	196	631	4584820	serine/threonine specific protein phosphatase
	213	233	199559777	serine/threonine-protein phosphatase 5
	213	126	22902393	thioredoxin reductase 1
	243	560	148677501	ATP synthase, H+ transporting, mitochondrial F1 complex, alpha subunit, isoform 1, isoform CRA_e
	253	333	148677501	ATP synthase, H+ transporting, mitochondrial F1 complex, alpha subunit, isoform 1, isoform CRA_e
	253	88	21314832	UTP—glucose-1-phosphate uridylyltransferase
	351	-	-	NO ID
	357	-	-	NO ID
	413	269	31982332	glutamine synthetase
	419	433	6754036	aspartate aminotransferase, mitochondrial
	419	149	123123581	heterogeneous nuclear ribonucleoprotein A3
	445	75	13385854	peptidyl-prolyl cis-trans isomerase D
	445	63	189339262	V-type proton ATPase subunit C 1
	474	146	14587839	acyl-CoA hydrolase
	516	362	148681931	capping protein (actin filament) muscle Z-line, alpha 2, isoform CRA_a
	573	64	122889678	ATP synthase, H+ transporting, mitochondrial F1 complex, gamma polypeptide 1
	637	41	29789148	NADH dehydrogenase [ubiquinone] 1 beta subcomplex subunit 9
	664	128	21312654	actin-related protein 2/3 complex subunit 5-like protein
	679	161	37700232	nucleoside diphosphate kinase A
	692	152	72004262	NADH dehydrogenase [ubiquinone] iron-sulfur protein 5
	692	73	18859597	NADH dehydrogenase [ubiquinone] 1 subunit C2
	692	77	6681095	cytochrome c, somatic
	701	339	156257629	beta-globin
	719	297	83715998	ATP synthase subunit e, mitochondrial
	729	232	19527388	ubiquitin thioesterase OTUB1
	783	332	6981602	syntaxin-binding protein 1
*P* <	100	981	163310765	serum albumin precursor
0.1	102	795	163310765	serum albumin precursor
&	116	368	40254595	collapsin response mediator protein 2
AR >	116	162	31560686	heat shock-related 70 kDa protein 2
|1.3|	663	-	-	NO ID
	693	424	6755040	profilin-1
	709	339	553919	alpha-1-globin
	711	329	156257629	beta-globin
	713	460	18655687	Chain B, Chimeric HumanMOUSE Carbomonoxy Hemoglobin (Human Zeta2 Mouse Beta2)
	769	36	6754086	glutathione S-transferase Mu 5
	777	65	17105370	V-type proton ATPase subunit B, brain isoform
	779	-	-	NO ID
	784	-	-	NO ID

**Table 3:** Variation in expression levels in enucleated (E) and anophthalmic (A) mice.

P value	Identification	SPOT	V1		V2M		V2L	
			E	A	E	A	E	A
P < 0.05	**Metabolism**							
	60 kDa heat shock protein, mitochondrial	191		↓				
	serine/threonine specific protein phosphatase	196		↑				
	serine/threonine-protein phosphatase 5	213						↑
	Thioredoxin reductase 1	213						↑
	ATP synthase, H+ transporting, mitochondrial F1 complex, alpha subunit, isoform 1, isoform CRA_e	243	↓					
	ATP synthase, H+ transporting, mitochondrial F1 complex, alpha subunit, isoform 1, isoform CRA_e	253	↓					
	UTP—glucose-1-phosphate uridylyltransferase	253		↓				
	aspartate aminotransferase, mitochondrial	419	↓	↓				
	heterogeneous nuclear ribonucleoprotein A3	419	↓	↓				
	peptidyl-prolyl cis-trans isomerase D	445	↓					
	V-type proton ATPase subunit C 1	445	↓					
	acyl-CoA hydrolase	474	↓					
	ATP synthase, H+ transporting, mitochondrial F1 complex, gamma polypeptide 1	573				↓		
	NADH dehydrogenase [ubiquinone] 1 beta subcomplex subunit 9	637		↑				
	nucleoside diphosphate kinase A	679	↓					
	NADH dehydrogenase [ubiquinone] iron-sulfur protein 5	692				↓		
	NADH dehydrogenase [ubiquinone] 1 subunit C2	692				↓		
	cytochrome c, somatic	692				↓		
	beta-globin	701				↓		
	ATP synthase subunit e, mitochondrial	719					↑	↑
	ubiquitin thioesterase OTUB1	729				↓		
	**Synaptic transmission**							
	N-ethylmaleimide sensitive fusion protein, isoform CRA_a	74	↑					
	vesicle-fusing ATPase	87	↑					
	syntaxin-binding protein 1	131	↑					
	syntaxin-binding protein 1	132	↑					
	glutamine synthetase	413		↑				
	syntaxin-binding protein 1	783	↑	↑				
	**Morphogenesis**							
	collapsin response mediator protein 2	74	↑					
	alpha-internexin	191		↓				
	capping protein (actin filament) muscle Z-line, alpha 2, isoform CRA_a	516						↑
	actin-related protein 2/3 complex subunit 5-like protein	664						↑
	**No identification**							
	No ID	124	↑					
	No ID	351	↑					
	No ID	357					↓	
p < 0.01	**Metabolism**							
&	serum albumin precursor	100					↓	
AR > |1.3|	serum albumin precursor	102				↓		
	heat shock-related 70 kDa protein 2	116				↓		
	alpha-1-globin	709				↓		
	beta-globin	711				↓		
	Chain B, Chimeric HumanMOUSE Carbonmonoxy Hemoglobin (Human Zeta2 Mouse Beta2)	713				↓		
							
	glutathione S-transferase Mu 5	769			↓			
	V-type proton ATPase subunit B, brain isoform	777			↓			
	**Morphogenesis**							
	collapsin response mediator protein 2	116				↓		
	profilin-1	693				↓		
	**No identification**							
	No ID	663						↑
	No ID	779				↓		
	No ID	784	↓			↓		

Identification of the protein spots by mass spectrometry was successful for 35 spots and allowed identification of a total of 36 distinct proteins. In V1 a total of 18 proteins were identified of which 9 were exclusively found in enucleated mice, 6 were exclusive to anophthalmic mice and 3 were found in both models of early blindness. In V2M, 13 proteins were identified and they were almost all found in anophthalmic mice (11/13). In V2L, 6 proteins were identified; 4 were only found in anophthalmic mice, 1 was exclusive to enucleated mice and 1 protein was found in both groups.

### Pathways affected by visual deprivation

Using the list of differently expressed proteins that were identified in V1, V2M and V2L of both enucleated and anophthalmic mice, IPA analysis revealed three pathways that were affected: metabolism, synaptic transmission and morphogenesis (Table 3).

#### Metabolism

Most of the proteins identified by the 2-D DIGE analysis are involved in metabolic processes (Table 3). The expression of these proteins was strongly affected by the loss of sight in both V1 and V2M, whereas it seemed almost unaffected in V2L. The majority of the differentially expressed proteins with a metabolic function was generally down-regulated.

Identified proteins were found to take part in the tricarboxylic acid cycle (TCA cycle; acyl-CoA hydrolase, spot 474; NADH dehydrogenase, spots 637 and 692; nucleoside diphosphate kinase, spot 679) required for ATP production, in the mitochondrial metabolism (cytochrome c, spot 692; ATP synthase mitochondrial, spots 243, 253 and 573) involved in energy production through the respiratory chain, in the regulation of oxidative stress (thioredoxin reductase 1, spot 213; glutathione S-transferase, spot 769), and in the proteasome activity (ubiquitin thioesterase, spot 729).

#### Synaptic transmission

Surprisingly, the 2-D DIGE screening technique revealed that only a few proteins involved in synaptic transmission were dysregulated at the adult stage (Table 3). Identified proteins were mostly involved in vesicle transport and fusion (N-ethylmaleimide sensitive fusion protein [NSF], spot 74; vesicle-fusing ATPase, spot 87; syntaxin-binding protein 1, spots 131, 132, 783) or in the glutamate cycle (mitochondrial aspartate aminotransferase, spot 419; glutamine synthetase, spot 413). Because the detection of membrane proteins is difficult with 2-D DIGE due to technical limitations [54–58], it is perhaps not surprising to find proteins involved in synaptic transmission that are not directly attached to the synaptic membrane. However, a direct link between the trafficking of GluA2 receptors and the NSF has been shown previously [59–65]. It is therefore possible that some membrane-associated proteins could also be differently expressed in enucleated and/or anophthalmic mice but remained undetected with the 2-D DIGE approach.

**Fig 2:**
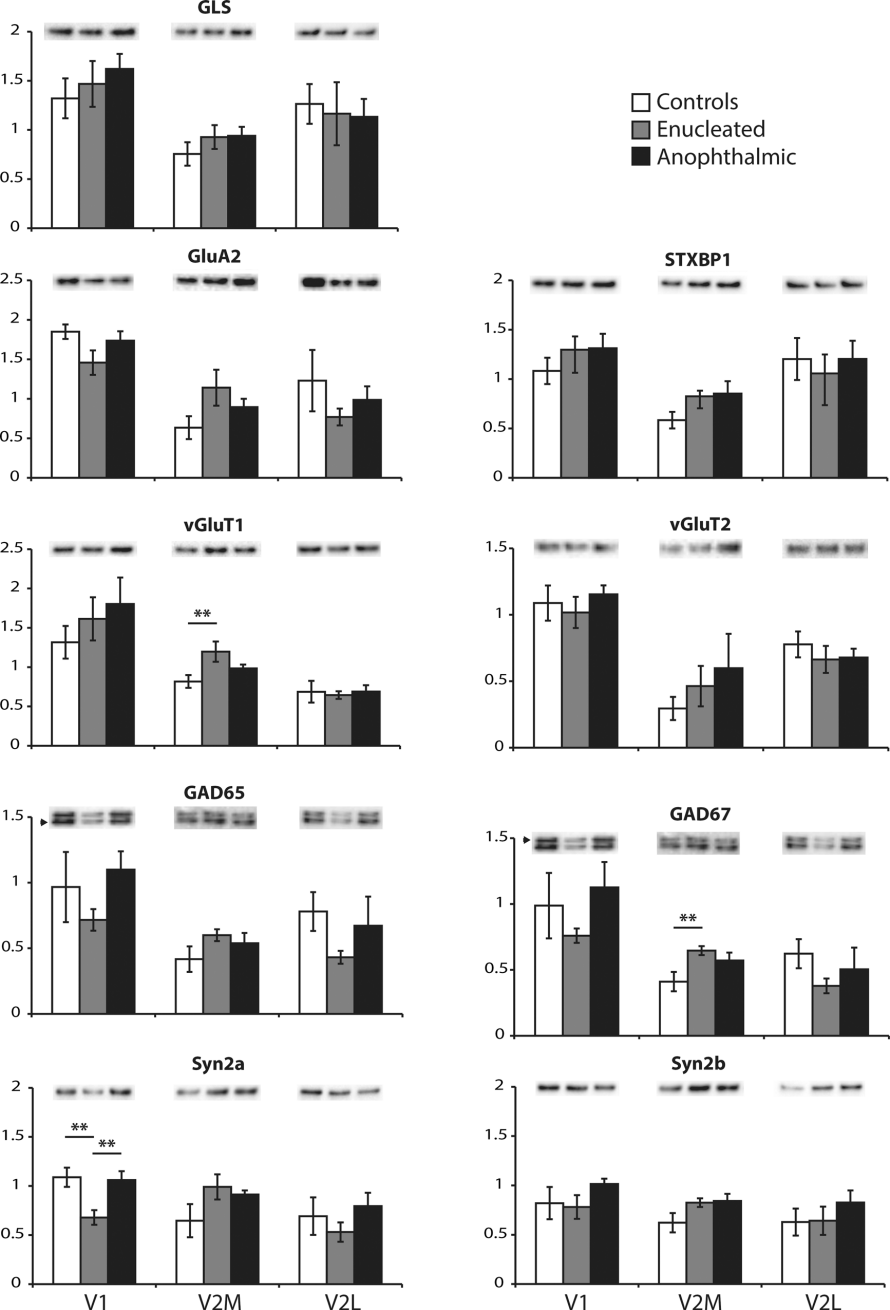
Proteins involved in synaptic transmission. The expression of glutamine synthetase (GLS), glutamate AMPA 2 (GluA2), syntaxin binding protein 1 (STXBP1), glutamate vesicular transporter 1 (vGluT2) and 2 (vGluT2), glutamate decarboxylase 65 kb (GAD65) and 67 kb (GAD67), synapsin 2a (Syn2a) and 2b (Syn2b) were analyzed by Western blotting. White bars are controls, grey bars are enucleated and black bars are anophthalmic mice. Results from V1 (left), V2M (middle) and V2L (right) are shown for each protein. ** *P <* 0.01.

To verify if this was the case, Western blot analyses were performed for proteins involved in synaptic transmission, including GluA2 (Fig 2). Of the 9 proteins tested, only 3 were significantly affected, and this only in enucleated mice. One protein, Syn2a, was down-regulated in V1 (Fig 2). No other changes were observed for all the proteins tested in V1, including GluA2. In V2M (Fig 2), only vGluT1 and GAD67 were affected and they were both up-regulated in enucleated mice. No changes were found in V2L for any of the tested proteins (Fig 2).

#### Morphogenesis

Proteins involved in morphogenesis were either up- or down-regulated and were detected for both blind mouse models (Table 3). Pathway analysis revealed axon outgrowth (collapsin response mediator protein 2 [CRMP2], spots 74 and 116) and actin polymerization and assembly (capping protein, spot 516; actin-related protein 2/3 complex subunit 5-like protein [ARP2/3 complex], spot 664; profiling-1, spot 693) as the main morphogenesis-related pathways.

**Fig 3:**
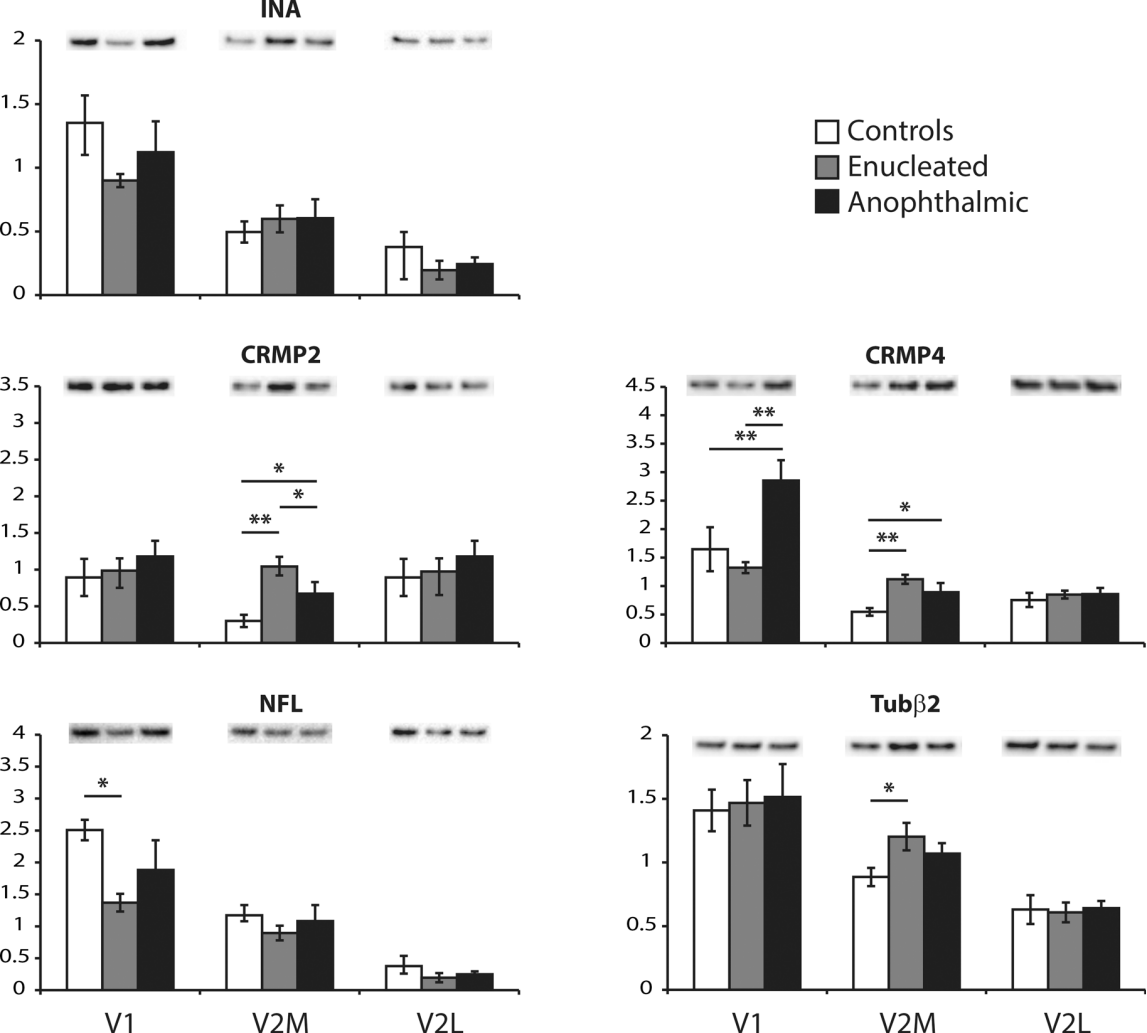
Proteins involved in morphogenesis. The expression of *a*-internexin (INA), collapsing response mediator protein 2 (CRMP2) and 4 (CRMP4), neurofilament-low (NFL) and tubuline-b2 (Tubβ2) were analyzed by Western blotting. White bars are controls, grey bars are enucleated and black bars are anophthalmic mice. Results from V1 (left), V2M (middle) and V2L (right) are shown for each protein. * *P <* 0.05 and ** *P <* 0.01.

In order to further investigate morphology-related protein expression changes occurring in visual areas of blind mice, Western blot experiments were performed for 5 proteins: alpha-internexin (INA), CRMP2, CRMP4, neurofilament-low (NFL) and TuBb2 (Fig 3). Choice was dictated by proteins identified with our 2-D DIGE analysis and by previous studies that identified proteins affected by visual deprivation (see [44] for Review). In V1 (Fig 3), the expression of CRMP4 was up-regulated in anophthalmic mice and NFL was down-regulated in enucleated mice. No changes were found for the other tested proteins in that area. In V2M (Fig 3), CRMP2 and CRMP4 were up-regulated in both enucleated and anophthalmic mice but TuBb2 was up-regulated only in enucleated mice. Expression of the other proteins was not affected by the loss of sight. No commensurate changes were found in V2L (Fig 3).

## Discussion

The aim of this study was to determine the long-term effects of the early loss of sight on the proteome in visual cortical areas of the mouse. 2-D DIGE was used as a screening technique to determine what pathways rely on visual input for their normal development by studying enucleated and anophthalmic mice, two models of perinatal and irreversible loss of sight.

Our 2-D DIGE analysis revealed that the expression of 36 unique proteins, out of 38 differential spots from V1, V2M and V2L are significantly altered by bilateral enucleation or congenital anophthalmia. The fact that some proteins were found in more than one spot indicates that different isoforms and post-translational changes were successfully detected by our screening technique. Overall however, the total number of identified differently expressed proteins is quite low considering that we screened three cortical areas from two blind mouse models with complete vision loss. In another study, 35 to 84 differentially expressed proteins were found in V1 of mice dark-reared from birth or from monocularly deprived mice and tested during or right after the end of the critical period [66]. In the present study, only 18 proteins were differentially expressed in V1. Although differences in technical procedures, mainly due to the low amount of membrane proteins that can be detected with 2-D DIGE [54–58], cannot be ruled out, the discrepancy in the number of spot most likely reflects the age of the animals used for the study. Indeed, in their study, the authors found more differences between P30 and P46 than between enucleated and sighted animals [66]. Other studies have also shown age to be an important regulator of protein expression. Investigations of mouse forebrain before, during and after the critical period revealed 251 differently expressed proteins [67]. In kittens, visual deprivation from eye opening until after the end of the critical period for ocular dominance plasticity resulted in protein expression levels [68] as well as molecular activity levels [69] indicative of a delayed maturation of visual areas. Moreover, they showed a return to normal values at a later stage [69]. This is consistent with another study also performed in cats showing that the expression levels of several synaptic receptors returned to normal values when visual deprivation, initiated at the time of eye opening, was maintained until after the end of the critical period and prolonged into adulthood [70]. In the present study, mice were visually deprived prior to (congenital anophthalmia) or from birth (binocular enucleation) until adulthood. It is likely that the maturation of the visual areas was initially delayed, with a larger set of proteins affected by the visual deprivation, but that protein expression levels gradually returned to normal values in later stages of post-natal development after the network had time to adjust to inputs from another sensory modality.

### Three pathways

Although few protein spots were differentially expressed, the 2-D DIGE analysis identified three pathways that were affected in the blind mouse models: metabolism, synaptic transmission and morphogenesis.

### Metabolism

Most of the differentially expressed proteins identified from our 2-D DIGE experiments were found to be involved in a metabolic pathway. All the observed changes in protein expression suggest that the production of ATP via the TCA cycle, the mitochondrial respiratory chain, the regulation of oxidative stress and the proteasome activity are slowed down in V1 and V2M of both blind mouse models and in V2L of anophthalmic mice. Considering that binocular enucleation and anophthalmia are two paradigms that completely deprive visual areas from their preferential inputs, it is perhaps not surprising that the main outcome is a decrease in metabolic activity. These results are also consistent with previous studies performed in monocularly enucleated rats [21] and monkeys with optic nerve transection [20], in which lower metabolic activity was revealed by a significant diminution in N-acetylaspartate levels, a metabolite that is linked to energy production by the TCA cycle and to mitochondrial activity [71]. Visual deprivation by lid suture, dark rearing and monocular enucleation also revealed a decrease in glucose metabolism in visual areas of rats [22].

Together with previous studies, the present results therefore indicate that the outcome of perinatal visual deprivation in mice, rats and monkeys that persists into adulthood results in lower metabolic activity in visual areas. This would suggest that the cross-modal inputs known to innervate [4, 10, 29, 37, 72–74] and to activate [2–4, 10, 72, 73] visually deprived areas are not sufficient to bring metabolic activity back to normal levels. This would support the hypothesis that cross-modal inputs are not optimally processed by an area normally associated to another sensory modality.

#### Synaptic transmission

Most of the changes in expression levels of proteins involved in synaptic transmission point toward the establishment of a new equilibrium between excitation and inhibition after a visual deprivation. In V1, the proteins that were differently affected indicate a decrease in glutamatergic transmission, possibly due to the loss of visual inputs into that cortical area, whereas both excitation and inhibition were affected in V2M, suggesting the establishment of a new equilibrium between remaining inputs.

The hypothesis that glutamatergic transmission is reduced in V1 of enucleated mice is supported by the downregulation of the mitochondrial aspartate aminotransferase, which synthesizes glutamate from the transamination of α-ketoglutarate in glutamatergic presynaptic terminals [75–77]. Furthermore, although the expression levels of both vGluT1 and vGluT2 were not affected in enucleated mice, the expression of Syn2a, which maintains the size of the vesicular pool at glutamatergic presynaptic terminals [78], was down-regulated. This suggests that the amount of glutamate per vesicle is unchanged but that the number of glutamatergic vesicles might be reduced after enucleation, potentially due to the reduction in glutamate synthesis. In anophthalmic mice, there was an increase in the expression levels of glutamine synthetase, which synthesizes glutamine from glutamate (see [79] for Review). Increasing its expression would also result in lower glutamate levels.

In extrastriate areas, only a few proteins involved in both GABAergic and glutamatergic transmission were affected in V2M and no changes were found in V2L. In V2M of enucleated mice, both GAD67, found in the cell body of GABAergic interneurons [80] and responsible for most of the GABA synthesis [81], and vGluT1, found in cortico-cortical synapses [82–85], were up-regulated. Alterations in the excitation/inhibition ratio can underlie the unmasking of cross-modal connections in humans [43] and changes in the excitation/inhibition balance are involved in the somatosensory reactivation of the visual cortex of monocularly enucleated mice [74]. V2M is known to be activated by cross-modal inputs in both enucleated and anophthalmic mice [2]. The establishment of a new balance between excitation and inhibition in enucleated mice [86, 87] could set a new threshold for cross-modal inputs [40–42], which could then be able to activate V2M. Based on the study of Chabot, however, one would expect changes in proteins involved in synaptic transmission also in V2M of anophthalmic mice but it was not the case here. Although it is possible that the different mouse strains used in both studies, i.e. the original ZRDCT/An strain versus our hybrid mouse strain, have distinct plasticity mechanisms [88], other mechanisms could also be at play in this blind mouse model.

#### Morphogenesis

In visual areas of sighted mice, there is a balance between excitation and inhibition (E/I) and also between visual and cross-modal inputs. In extrastriate areas, multisensory neurons have been identified [89], whereas cross-modal inputs mostly have a subthreshold influence in V1 [40–42]. After binocular enucleation and anophthalmia, the complete loss of visual inputs disrupts both the E/I balance and the equilibrium between visual and cross-modal inputs. As they are the only remaining source of inputs in the visual areas of blind mice, cross-modal inputs could be potentiated.

V2M was the area where most proteins involved in morphogenesis were affected. The expression of CRMP2 and CRMP4 were up-regulated in both enucleated and anophthalmic mice, whereas Tubβ2 was up-regulated only in the latter. CMRPs are important mediators of the semaphorin pathway [90–92], which promotes axon growth cone collapse, and are also involved in microtubule dynamics [93–95]. CRMP2 and CRMP4 respectively co-localize with pyramidal neurons and inhibitory interneurons [96]. The high axonal dynamics could indicate rewiring of cortical circuits that result in a network that is potentially more favorable for cross-modal inputs.

In V1 of enucleated mice, a reduction in NFL expression was found. This protein is known to affect the diameter of the ventral root axons [97, 98] and of axons in the brain, cervical spinal cord and sciatic nerve of the mutant of the Japanese quail [99–102]. Our results therefore suggest a reduction in axonal caliber, which could be directly linked to the loss of visual inputs. In anophthalmic mice, the increase in CRMP4 levels that was found in V1 suggests the presence of shorter and simpler inhibitory axons [96], which could induce a reduced inhibition on preexistent cross-modal inputs [29]. In V1, the potentiation of non-visual inputs could therefore occur through the pruning of visual fibers in enucleated mice and through disinhibition of cross-modal circuits in anophthalmic mice, a phenomenon also believed to occur in blindfolded humans [43].

### Enucleation vs anophthalmia

In this study, bilateral enucleation and congenital anophthalmia affected the expression of a distinct set of proteins that were involved in metabolism, synaptic transmission and morphogenesis. Binocular enucleations were performed at P0. At that age, retinal axons have reached the dorsal part of the lateral geniculate nucleus (dLGN, at E13) and the thalamocortical axons have reached the subplate (at E16), but have not yet reached layer IV [103]. Through spontaneous retinal waves that occur during embryonic development, patterned visual information can be carried from the retina to the dLGN and to the subplate to promote cortical development prior to birth [104]. This information is required for the establishment of the normal retinotopy [105, 106] and of the segregation of retinal afferents in subcortical and cortical structures [106–111]. Removing the eyes at P0 therefore allows for normal visual circuits to develop but prevents patterned visual innervation of layer IV in V1. In the present study, anophthalmic mice were obtained by backcrossing ZRDCT/An mice with C57BL/6J mice. Congenital anophthalmia in ZRDCT/An mice is caused by a mutation of the Rx/Rax homeobox gene [112], which prevents the development of the optic nerve and optic cup [113] and, therefore, results in a complete absence of visual inputs during embryogenesis. This mutation is also responsible for congenital anophthalmia in humans [114, 115].

In hybrid mice similar to the ones used here, it was shown that the volumes of V1 and of the dLGN were significantly smaller in enucleated and anophthalmic mice than in sighted control littermates, but not different between both types of blind mice [116]. Furthermore, very few changes in the number of cross-modal connections onto V1 were found in both enucleated and anophthalmic mice with respect to sighted controls [29]. Both visual deprivation paradigms were however found to alter the establishment of callosal connections [117–124] and of the striate-extrastriate projections [124–126]. The topographic anomalies were greater in anophthalmic than in enucleated mice [124]. At the subcortical level, ectopic connections from the inferior colliculus to the dLGN have been described in ZRDCT/An but not in enucleated mice [23]. Re-wiring of retinal axons on the medial geniculate nucleus of the thalamus in ferrets results in auditory neurons with visual properties [127–129] and a normal pattern of cortico-cortical connections [130]. Similar results were obtained after neonatal bilateral ablation of the superior colliculus in hamsters [131]. If the visual areas of anophthalmic mice, which never received visual inputs, are targeted by ectopic subcortico- and cortico-cortical auditory and potentially also somatosensory afferents, they could in fact process non-visual information. This could explain why very few changes were found for proteins involved in synaptic transmission and morphogenesis in these mice. This is in line with results from congenital anophthalmic humans in which the loss of visual inputs affects structures at the subcortical level and to a lesser extent the Calcarine sulcus, whereas the rest of the cortex appears unaffected [132]. Furthermore, the lack of competition between visual inputs and other sensory inputs could also limit the effect of anophthalmia on these two molecular pathways. This would be consistent with other studies that have shown that the complete lack of sensory inputs from one sensory modality might be less detrimental than a partial loss [133–135]. In enucleated mice, the broader changes in the three identified molecular pathways could indicate the involvement of homeostatic and cross-modal plasticity mechanisms in order to compensate for lack of subcortico-cortical inputs. In both models of early blindness, the proteins involved in metabolic pathways that were differently expressed suggest a lower energy metabolism in visual areas. This could be due to the fact that they now process non-visual stimuli, but in a non-optimal manner.

### Differential effect in extrastriate visual areas

Our results demonstrate that most changes occur in V1, which is not surprising considering the predominant visual function of this cortical area. A more unexpected result was that V2M is particularly affected by the loss of visual inputs, whereas V2L displays very limited changes. The reason why V2M is more affected than V2L is not clear based on anatomical evidences as both areas receive cross-modal inputs. Indeed, direct somatosensory inputs were shown to promote the reactivation of the medial monocular visual areas after monocular enucleation in adult mice [72] and V2L was shown to participate in an indirect connection between the primary auditory cortex and V1 [37]. Although both V2M and V2L have a similar potential for multisensory processing in rodents [89, 136], auditory stimulation has been shown to activate V2L and V2M in bilaterally enucleated mice [2] and V1 and V2M in anophthalmic mice (ZRDCT/An strain [2, 3]). It is therefore possible that V2M has a greater potential to process new sensory stimuli than V2L. This would be consistent with the observed cross-modal recruitment of V2M and open-eye potentiation in V2L after monocular enucleation in adult mice [72]. The exact reason remains unclear but it could be linked to the fact that topographic projections from V1 to medial extrastriate areas are less well defined than the ones to lateral areas [124, 137].

## Conclusion

Surprisingly few permanent protein expression changes characterized the visual cortex of adult mice that underwent a perinatal visual deprivation compared with sighted mice. This rather modest molecular footprint could indicate that cross-modal inputs into visual areas are sufficient to bring cortical activity levels back to a baseline level very similar to what is found for visual inputs in sighted mice. Although the nature of the inputs themselves is different, cortical neurons are activated and the cortical excitation/inhibition balance could be adjusted accordingly. The changes in metabolic activity nevertheless seem to mirror a suboptimal processing of the cross-modal inputs in visual areas of blind subjects.
